# Visual fields interpretation in glaucoma: a focus on static automated perimetry

**Published:** 2013-06-04

**Authors:** Moustafa Yaqub

**Affiliations:** Professor of Ophthalmology, Assiut University, Egypt and Head of Ophthalmology Department, Royal Care International Hospital, Khartoum, Sudan.

**Figure:**
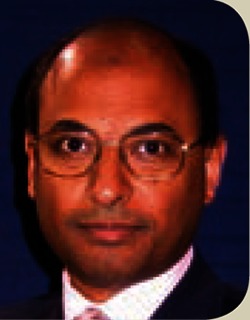
Moustafa Yaqub

Examining the visual fields is an integral part of any ophthalmic evaluation.

All ophthalmologists ordering and interpreting visual fields should go through the experience of having visual field tests done on them, and also perform some tests on patients themselves. This may help them better understand and use the different perimeters available, and improve their communication with their technicians.

There are so many excellent textbooks covering the subject of visual fields in glaucoma, and the reader is advised to go through at least one of those to grasp the whole topic. This article focuses on some practical aspects of visual field testing, including patients with multiple pathologies, examining the patient with advanced glaucoma, and the differential diagnosis of glaucomatous field defects, **This article addresses the use of the ‘Humphrey’ perimeter, but also includes brief notes on the Octopus printout**.

Before starting, certain facts must be remembered:

1. Manual and/or automated VF plotting is a **subjective** test. So, if you get meaningless results, it could be due to improper subject selection, or poor patient understanding of how to perform the test.
2. The VF reflects changes in the visual pathway. It does not diagnose glaucoma. One should always look carefully at the fundus, to see if the VF defects match the appearance of the disc and retina or not. The presence of neuro-retinal rim pallor, for instance, excludes glaucoma as the **only** pathology responsible for the field defects (even in the presence of a confirmed glaucoma diagnosis).
3. While visual acuity is the single most important disease progression parameter for patients, and visual fields are still the most important disease progression parameter for physicians, the two tests cannot be separated when one looks at the visual function and the functional reserve of the eye. After all, our patients are concerned with what they see, without separating their visual acuity from their visual fields. Like most glaucoma specialists, I have had cases of advanced glaucoma where only a temporal crescent remains. Those patients have developed ways of using their existing field, and have carried on using it to perform life activities. It is our duty to help them keep their functional vision, despite the extensive loss.

## Terminology

Two similar terms are commonly used:

1. **Field of view.** All physical objects and light sources that form images on the retina at any particular moment, while fixation is fixed.
2. **Visual field.** The perception of those objects and light sources by the visual cortex, after being processed by the visual pathway. This is subject to individual psycho-physical factors.

**Figure 1:**
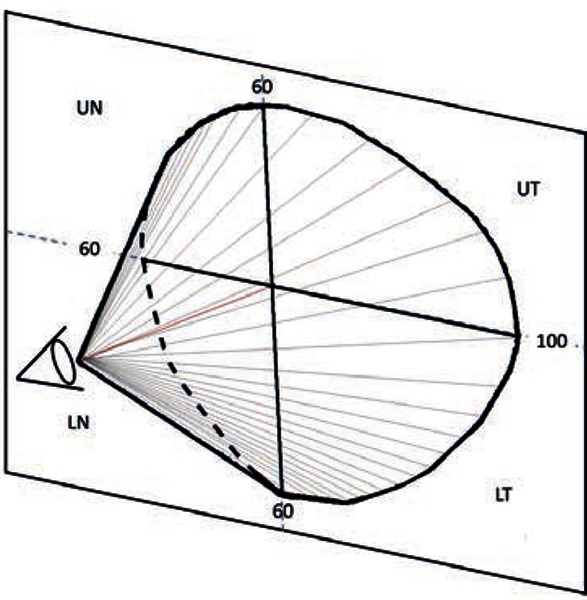
The three-dimensional conical structure of the visual field. For the right eye, the boundaries of the field are 100 degrees temporal and 60 degrees upper, lower, and nasal. The apex of the cone is at the nodal point of the eye, where light rays converge on their way to the retina, forming the perimetric angle.

Normally, these two terms may be used synonymously. However, in some pathological conditions, certain areas of the field of view may not be perceived (seen), and a field defect results. These defects are either “relative” or “absolute,” depending on the degree of damage caused by the pathology, and the remaining functional reserve of the visual system.

In primary open angle glaucoma (POAG), the development of these defects is usually slow, and may be masked by the overlapped visual fields of both eyes to produce a single binocular field. Indeed, up to 40% of the retinal ganglion cells may be lost, before any VF changes could be detected.

The presence of more than one pathology affecting the visual pathway (with superimposed multiple defects) may complicate our interpretation of field testing. This is important, since most patients diagnosed with POAG present at an age where by other factors may also have an effect on the visual apparatus.

## Visual field boundaries

The VF is a three dimensional cone (Traquair's Island of vision), with its apex at the nodal point of the eye, and its base at infinity (or at whatever distance we plot it by a perimeter screen or bowl) (Figure 1). The purpose of visual field testing is to define the topography of the island of vision to recognize any variation from normal.

**Figure 2:**
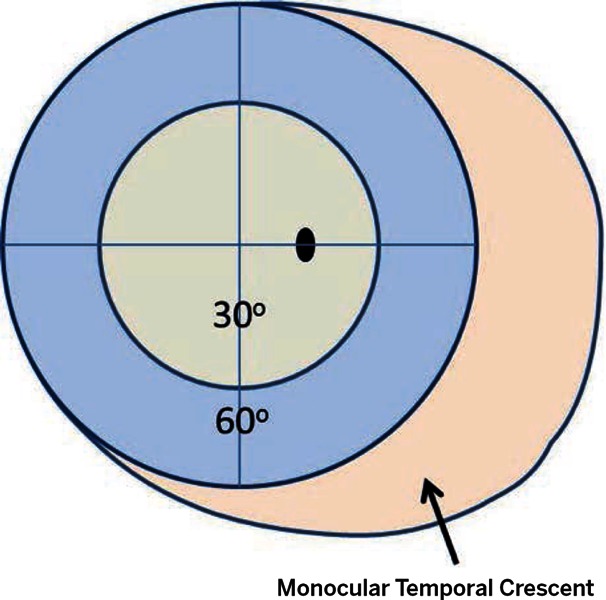
Diagram of the visual field for the right eye. The three regions of the field are the central field (0 to 24 or 30 degrees), the peripheral field (30 to 60 degrees), and the temporal crescent.

The plotted VF (the base of the cone) extends for approximately 60 degrees superior, inferior, and nasal and 100 degrees temporally. For practical purposes, the VF plot may be divided into three major parts: the central 30 degrees, the peripheral field (from 30 to 60 degrees), and the temporal crescent (Figure 2).

**Figure 3:**
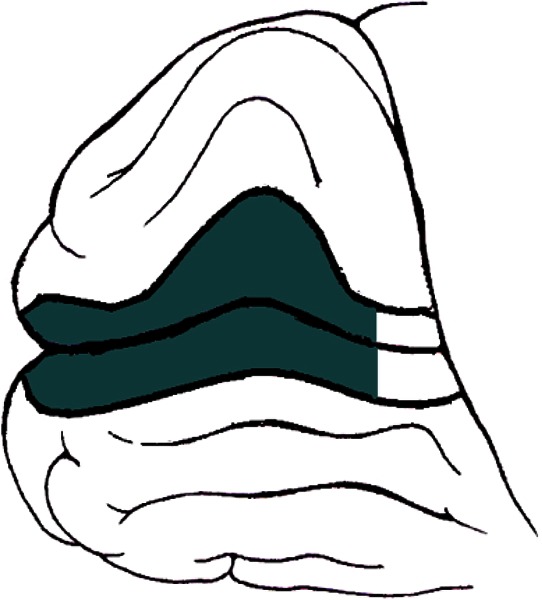
Cortical representation of the central visual field. The central 30 to 40 degrees of the visual field occupies 83% of the striate cortex.

In the large part of our practice today, automated static perimeters are used to test the **central VF**. The central VF reflects the function of approximately 66% of the retinal ganglion cells, and its cortical representation occupies 83% of the visual cortex. Thus, exploring it could reveal almost any pathology in the visual pathway (Figure 3).

These days, the need to explore the **peripheral visual field** is limited to searching for a ring scotoma, confirming a nasal step defect, and maybe looking at the temporal crescent. These defects can easily be discovered using a tangent screen and/or careful confrontation techniques. Investing in an ultramodern automated kinetic perimeter may thus be avoided, especially in the face of limited resources.

## Visual field centre

The horizontal axis of the visual field is mostly represented by the horizontal raphé of the retina. The vertical axis, however, is represented by two boundaries, a short vertical line through the foveal area (fibres nasal to it go to the nasal half of the disc), and a semi-vertical line through the centre of the disc, separating the remaining nasal fibres from the temporal fibres of the retina (Figure 4).

This fact should always be remembered, since it explains the reason for “Band Atrophy” of the optic disc in lateral geniculate body lesions, which also produce VF defects (sectoranopias) mimicking glaucomatous defects.

**Figure 4a:**
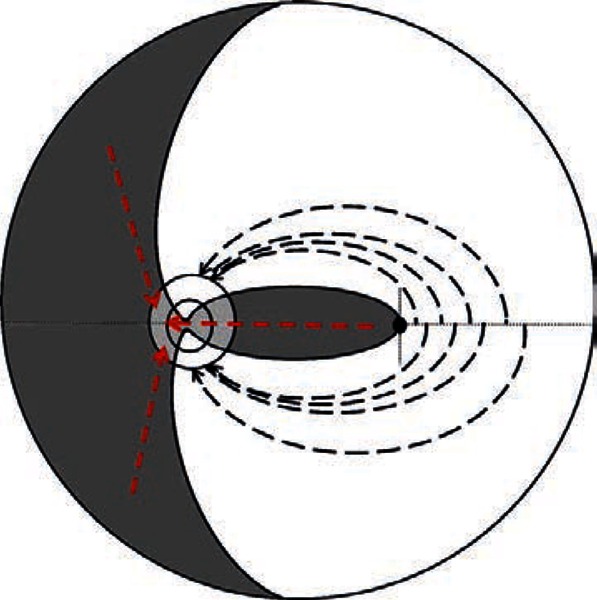
Orientation of retinal ganglion cells axons in the nerve fibre layer of the left retina. At the fovea, a vertical line separates the narrow band of nasal fibres (which join the nasal half of the optic disc), from the temporal arcuate fibres which join the superior and nferior poles of the optic disc. The horizontal raphe is at the horizontal axis. The light grey areas on the disc show the development of Bow-Tie (Band) atrophy in Chiasmal lesions. In this case the pallor will involve both the neuroretinal rim and the cup at the 9 and 3 o'clock positions.

**Figure 4b:**
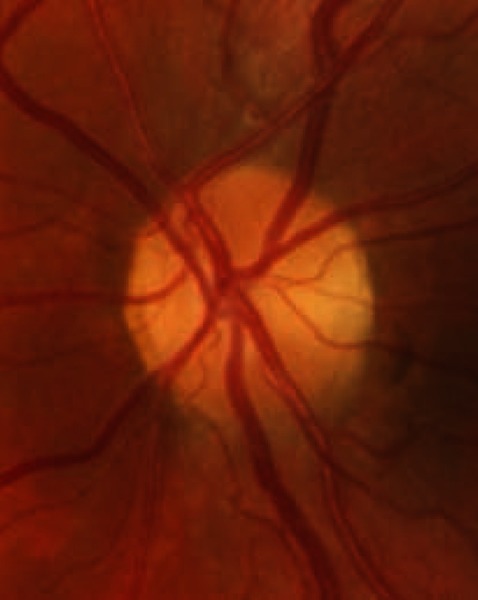
Band atrophy of the optic disc.

## Examination strategies

Static perimeters test the differential light sensitivities of specific retinal locations distributed on a fixed grid pattern. The spacing between these locations (points) varies according to the examination area targeted. When testing the central 30 degrees and central 24 degrees with the Humphrey perimeter, the grid points are spaced 6 degrees apart. The central horizontal points may be distributed on the horizontal axis (programme 30-1 or 24-1), or at equal distances from the horizontal axis (programme 30-2 or 24-2). The advantage of using the central 30-2 or 24-2 programmes lies in the spacing of the central horizontal points at 3 degrees from the centre of the field, with greater sensitivity to changes across the horizontal retinal raphé (nasal steps) (Figure 5).

**Figure 5a:**
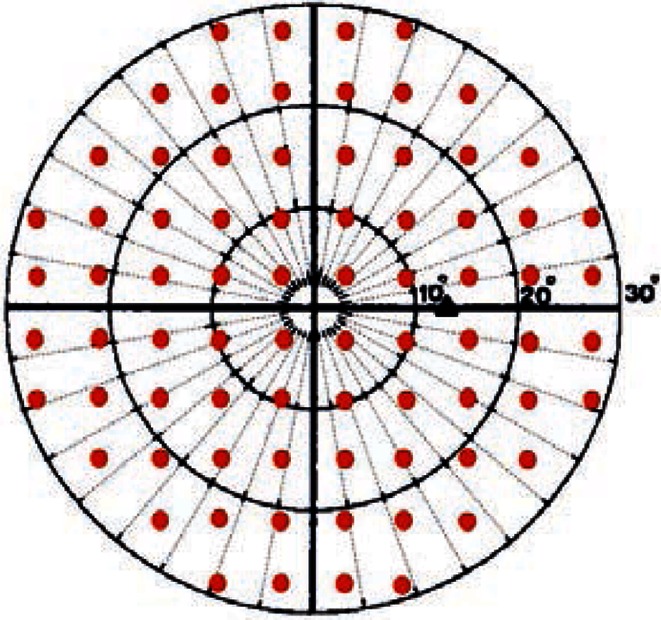
The central 30-2 programme. Note the distribution of the horizontal test spots at equal distances from the horizontal axis (3 degrees). This insures more detailed exploration of horizontal defects (nasal steps). Note also the small number of test locations in the central 10 degrees. Compare this with the number and distribution of test locations in the central 10-2 programme.

**Figure 5b:**
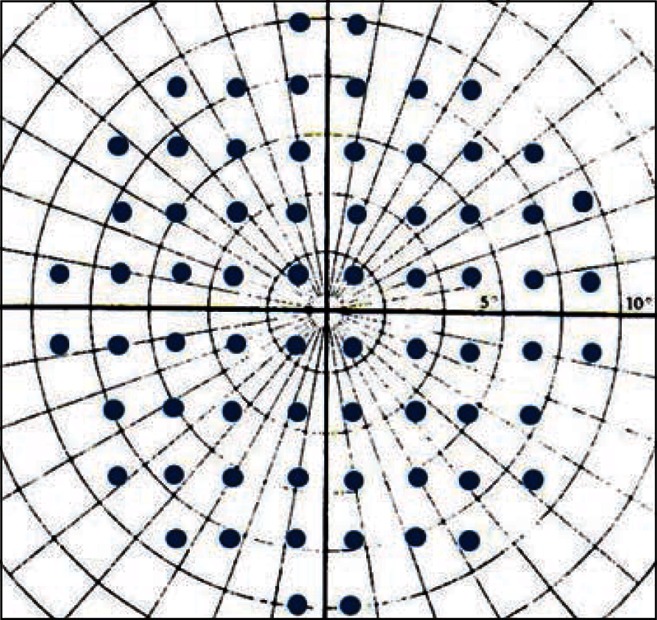
The central 10-2 programme. The space between test spots is 2 degrees. The horizontal test locations are distributed at 1 degree from the horizontal axis.

**Figure 6a:**
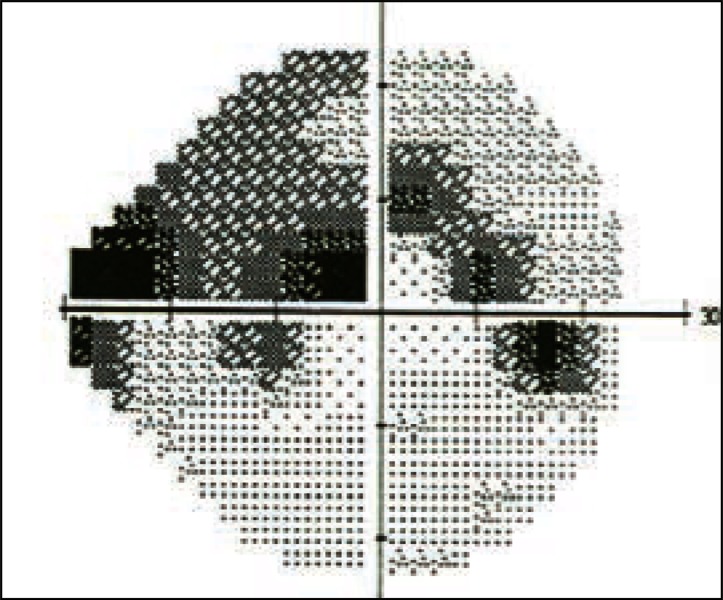
A central 30 degree field for advanced POAG. This grey scale shows a deep central defect encroaching on fixation.

**Figure 6b:**
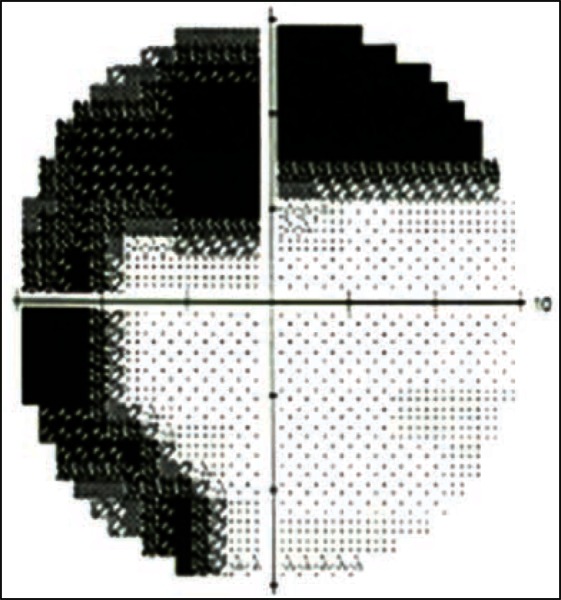
Using the central 10-2 programme for the same eye shows that the defect has not crossed fixation yet.

**Figure 7:**
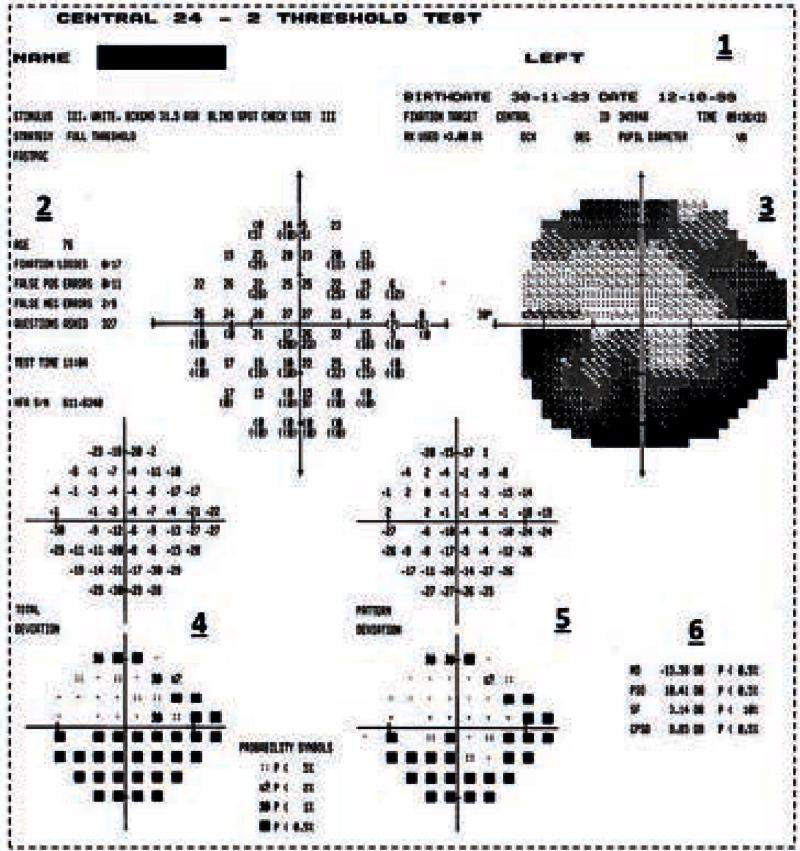
Sequence of examining the printout. 1. Patient's data and test used. 2. Reliability indices. 3. The grey scale (which must be examined with the other eye's grey scale at the same time). 4. The tota deviation. 5. The pattern deviation. 6. Global indices.

One of the most useful programmes, particularly in advanced glaucoma, is the central 10-2 programme. The test locations are 2 degrees apart, and a large number of points are crowded in the central 10 degrees. This gives a magnification effect, and shows the relationship between the central field defects and the point of fixation more precisely. This may reflect on the decision to operate or not, and helps with estimation of the risk of postoperative wipe-out (Figure 6).

## The Humphrey printout

**Figure 8:**
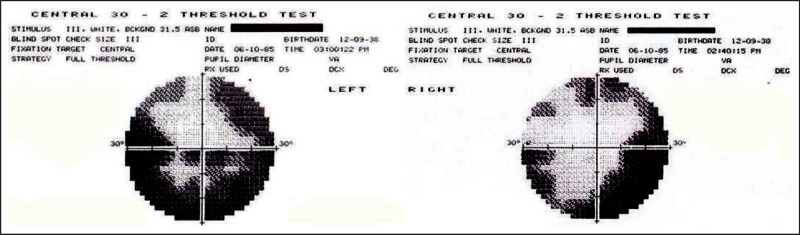
Examining the central VF grey scale of the right eye alone may be confusing, as there is no specific pattern to the field defect. However, looking at the two grey scales together reveals a left central field defect, signifying optic neuritis. In this case, looking at the rest of the printout data (statistical package) may be useless, as it is designed to analyse glaucoma defects only.

The test results (with or without the statistical analysis) are usually printed on one sheet called the printout (Figure 7). When examining the test results, you MUST hold the two printouts (right eye print out in your right hand facing your right eye, and left eye printout in your left hand facing your left eye), and examine them together (Figure 8).

### Patient's data and test parameters

These are at the top of the printout.

- Your first priority is to make sure that those fields belong to the patient in question, and that the specific program requested has been used, with the parameters that you have asked for.
- The date of birth has to be correct (for comparison with range of normal values for age). A pupil diameter of at least 2.5 mm is essential to avoid overall depression of test values. Finally, using near correction lenses, and/or high astigmatic error correction lenses is strongly advisable to help the patient appreciation of the test targets. A frameless lens may be best suited for that, but other lenses could be used and allowance for any rim artefacts made during interpretation.

**‘When examining the test results, you MUST hold the two printouts (right eye print out in your right hand facing your right eye, and left eye printout in your left hand facing your left eye), and examine them together’**

### Reliability indices

The second priority is to check your patient' performance (reliability). These parameters are printed at the top left hand side of the prin out In the Humphrey perimeter, and at the bottom right hand side for the Octopus perimeter.

- Fixation loss. Normally this is between 0 to 2%. If the loss exceeds 20%, this is generally considered as poor reliability. However, it may also be an indicator of advanced glaucoma with an abnormally large blind spot. Other indicators of advanced glaucoma should be looked up first, before disqualifying the test results as unreliable based on poor fixation.
- False-positive or false-negative responses. Scores in excess of 20-30% indicate a test of questionable reliability.

### The grey scale

- This is a graphic representation of the recorded threshold sensitivities in the numeric scale. Regions of decreased sensitivities are displayed in darker tones. The grey scale plot is very useful for displaying patterns of loss (nerve fibre layer defects versus neurological defects) and thus should be inspected first. When looking for such patterns, both right and left grey scales should be inspected together. If any visual field defect related to pathology of the visual pathway distal to the lamina cribrosa is revealed, then attention is directed to other tests to examine the rest of the visual pathway.
- It is also a very useful tool in explaining – to the patient and family – the stage of the disease and its progress over time.

### The numeric scale

This is the main test result. It shows the retinal sensitivities at the different test locations, expressed in Decibels (dB). In the Humphrey perimeter, 10 points are retested, and in those points, the two results are printed next to each other. This is done to determine the short term fluctuation (see later). In the Octopus perimeter, retesting is done in **all** test locations.

The numbers expressed in the numeric scale may be looked at as heights, and with the higher values in the centre, and the least values at the periphery, one can ‘see’ the centre of the hill of vision in a three dimensional way.

## Statistical analysis of test results

Inclusion of statistical analysis software is the reason for the widespread popularity of static perimetry. Computers can store a huge amount of numbers and use them to look for patterns, compare them with stored data bases, and perform all kinds of analysis on them.

The Statpac software is a commonly used analysis tool in both Humphrey and Octopus machines. It is used to look at suspicious clusters of numbers, analyse them, and monitor their change over time.

### Total deviation plot

- These numbers show the difference (in dB) between the test results and the normal values expected for the patient's age group. 0 dB = no difference (normal), while −13 dB = large depression from normal value (see the total deviation plot in Figure 7). The true value of the defect in this instance should be −17 dB, but the computer allows for a variability of −4 dB.
- This is in fact a plot of the **probability** of each point change being normal. If less than 0.5% (indicated by the solid black squares), then the point change is highly unlikely to be normal.

### Pattern deviation plot

This particular analysis tool is helpful for detecting visual field defects (scotomata) in the presence of media opacities, such as cataract. It does this by looking at the overall **sensitivity changes** in the hill of vision (the ‘pattern’ here is the conical shape of the hill of vision). If there is an overall depression (all test values are reduced from normal due to cataract), then it will subtract this value from all test points, leaving behind clustered field loss (localised defects), which may be due to glaucoma.

**‘Inclusion of statistical analysis software is the reason for the widespread popularity of static perimetry’**

## Global indices

These numbers represent mathematical summaries of all the sensitivity values produced by the test. They are useful tools for having a quick idea about the entire field, and sequentially compare test results for the same eye (change analysis). Looking at them however, does not replace examining other test data in detail. Note that the newer SITA (Swedish Interactive Threshold Algorithm) software has just a pattern standard deviation. A useful article can be downloaded here: http://tinyurl.com/ visfieldSITA (PDF, 240kb)

### Mean deviation

This number reflects the overall depression (deviation from normal values) of the field. All the obtained values of the test are added, and divided on the number of test locations. This gives the mean value of the test. The same is done for the normal expected values stored in the computer data base. The difference between the 2 values represents the MD. Normally it should not exceed −2 dB. If the MD is significantly outside the normal, then a P value is assigned to it.

### Pattern standard deviation

For practical purposes, the pattern standard deviation (PSD) reflects the degree of departure (difference) of the measured VF pattern (shape) from the normal hill of vision. A small PSD reflects a smooth uniform hill of vision, while a large PSD value reflects an irregular hill of vision (Figure 9).

**Figure 9a:**
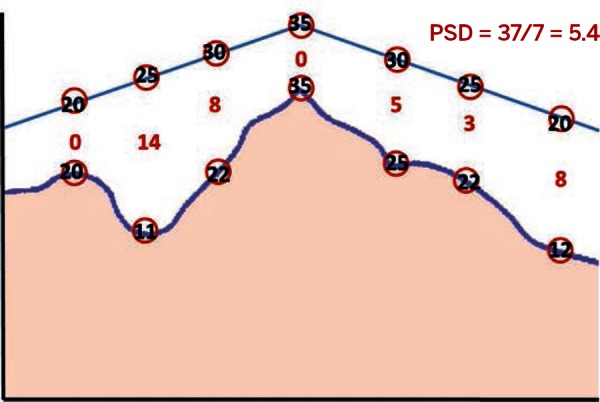
The pattern standard deviation (PSD) is the difference between the expected data (the straight line at the top) and the patient's own data (the irregular line beneath it). Here, the sum of the differences is 38, which, when divided by number of test locations, yields 5.4. This reflects an irregular hill of vision.

**Figure 9b:**
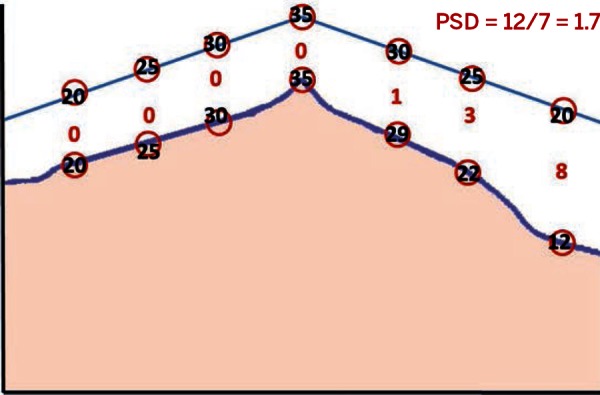
The sum of the differences is here is 1.7, a much smaller number, which reflects a much smoother hill of vision with a more normal pattern to the visual field.

### Short-term fluctuation

The short-term fluctuation (SF) value reflects difference in the response for certain test spots on re-testing. It is used to refine the test results by excluding errors due to patient fatigue. Normally it should not exceed 2 dB. However, high SF may be a sign of early glaucoma.

### Corrected pattern standard deviation

An irregular hill of vision revealed by a high PSD value may be due to low patient reliability, actual VF defect, or both. By removing patient fatigability factors (correcting the PSD by subtracting SF value), the true shape of the hill of vision is appreciated; this is the corrected pattern standard deviation (CPSD) (Figure 10).

## The change analysis printout

This is a simple graphic representation of summaries of sequential VF tests for the same eye using all global indices. It allows you to track changes over time, and evaluate the progression (or stability) of the glaucomatous process. Care should be taken when using change analysis, because it requires many reliable fields (at least 4 excluding the first one to avoid learning mistakes). To compare results from a small number of fields, one can use the ‘Multiple fields printout’, to visually track changes between the tests in question.

**‘The change analysis printout allows you to track changes over time’**

## The glaucoma hemifield analysis

This software allows comparison of VF defects across the horizontal axis (looking for – and comparing – nasal steps). As such it alerts you to the need to re-examine the printed results, looking for such differences. The three important responses to look for are:

1. Within normal limits (no differences)
2. Borderline (early differences)
3. Outside normal limits (obvious differences between the upper and lower halves of the field)

In most instances, such differences are seen in the other graphical plots.

## Notes on the Octopus printout

Data representations in the Octopus printout are – for the most part – similar to those in the Humphrey printout. Patient and examination data are at the top of the printout, followed by the value table (numeric scale) and the grey scale (which is coloured). These are followed by a comparison table and a corrected comparison table (total deviation and pattern deviation). The probability plots for those tables follow. Finally the visual field indices (global indices) are at the bottom right hand side of the printout page.

**Figure 10a:**
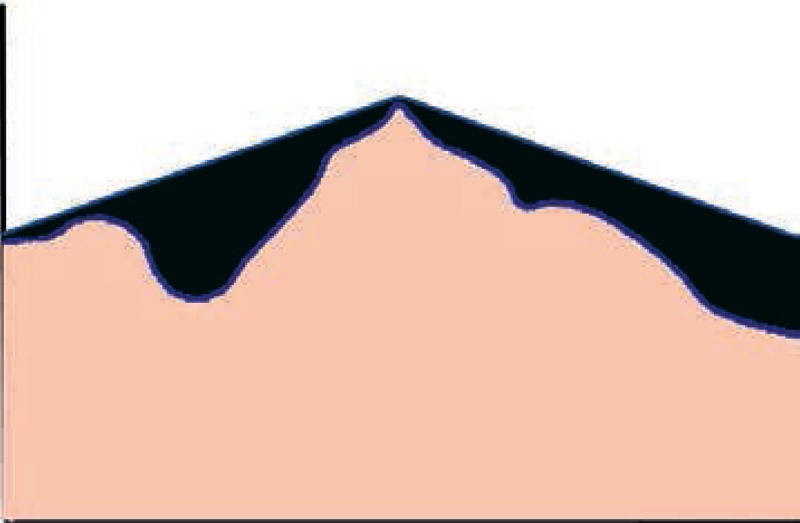
Removing the effect of the short term fluctuations (SF), reveals the true difference (in green) between the regular “expected” pattern of the hill of vision, and the patient's hill of vision.

**Figure 10b:**
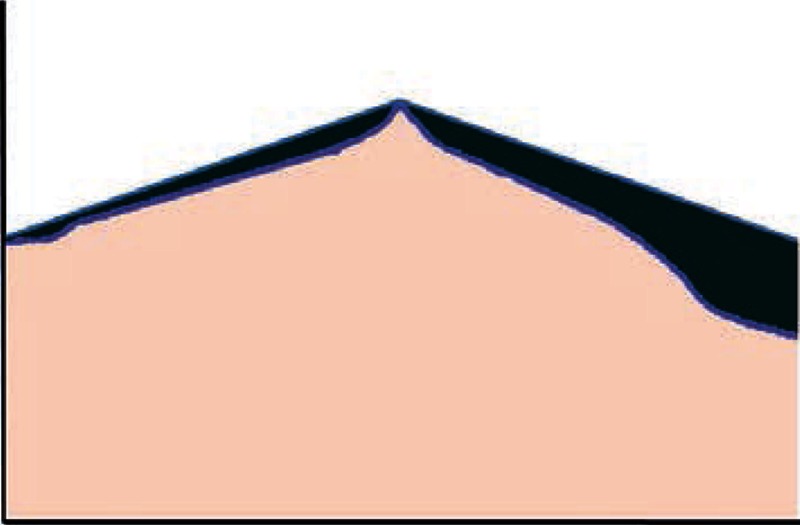
The difference here is small, indicating a smoother hill of vision (closer to normal pattern).

The indices are MS (mean sensitivity) which is the mean value of all test results, MD (mean defect) which is the same as mean deviation, LV (lost variance) which is the same as pattern standard deviation, and CLV (corrected lost variance, which is the same as corrected pattern standard deviation). The RF (reliability factor) is a score of patient's reliability (0 to 15%).

The major differences between the two printouts are the presence of the Bebie curve, and phasing.

**Figure 11:**
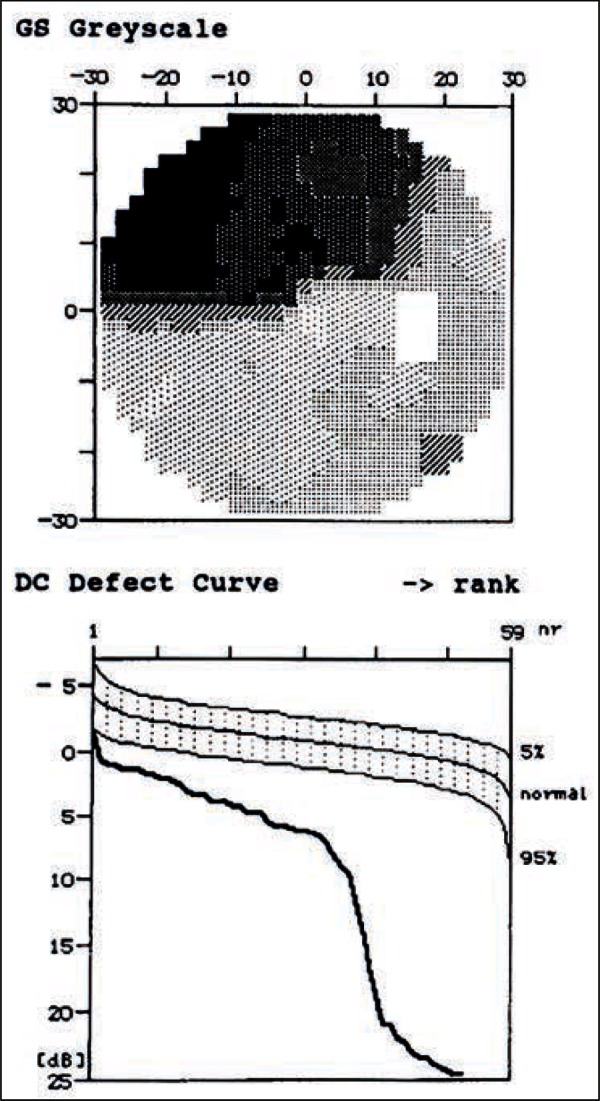
This cumulative defect curve shows both generalised depression of the field values, as well as a large deep localised defect (seen in the grey scale). It doesn't however signify the type of the defect.

The Bebie curve is a cumulative defect curve that allows ranking of VF defects (it looks only at the defects, not the field). In the presence of established glaucoma diagnosis, this is useful to monitor the progression of both overall depression and localised defects (Figure 11).

Staging VF testing is a useful feature. It allows collecting examination results from points considered most important for glaucoma diagnosis in the first and second stages (80% of the test). This allows printing of these results and stopping the testing process, in case of patient fatigue. In all other perimeters, the entire testing process has to be completed before printing of the results.

## Monitoring visual fields in advanced glaucoma

Two major problems arise in patients with advanced field loss in glaucoma:

1. **Threat to fixation.** The central progression of VF defects towards fixation is an ominous sign of progression. Most surgeons fear this because it may be related to a higher risk of visual loss following surgery for glaucoma. To allow for better interpretation of these fields, one must use the Central 10-2 program available in almost all threshold testing perimeters. This allows “Zooming in” and magnification of the central field, revealing the true relationship between field defects and fixation point. This may allow for better planning for future surgeries in advanced glaucoma (Figure 6).
2. **Depression.** As a progressive blinding disease, the psychological impact of glaucoma on patients cannot be overemphasised. This affects their performance in VF testing, as well as their compliance with medications, and their quality of life. To help patients perform better in VF testing, and improve their moral, one may use a larger target size (s ize IV) for testing.

**Figure 12a:**
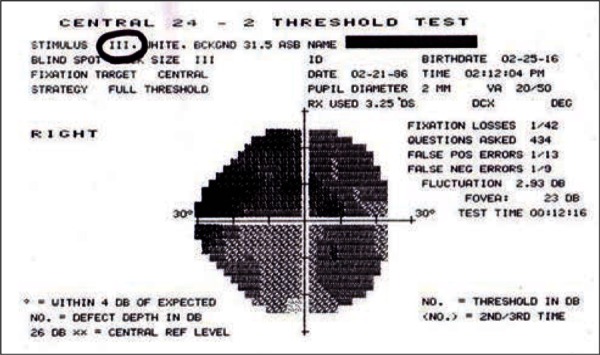
VF using a size III spot size.

**Figure 12b:**
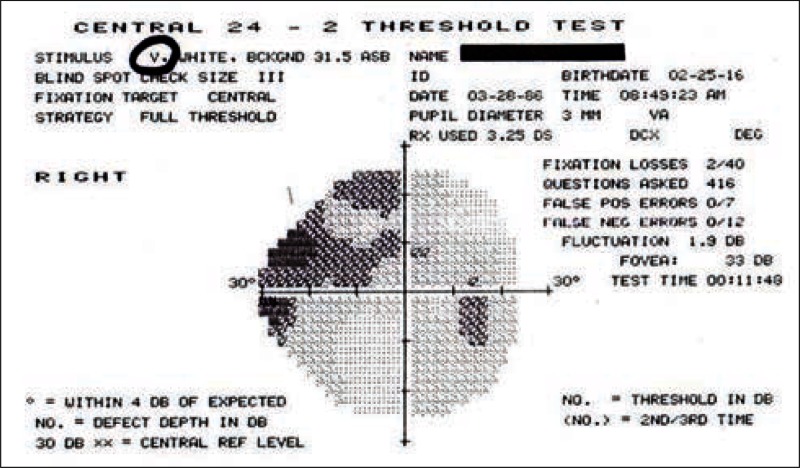
Same VF with a size IV spot size. This can be used as a new baseline for subsequent examinations, and is better for patient's morale.

The standard size III in SAP (standard automated perimetry) is mainly used for easier and faster mapping of the blind spot. Its use in all VF testing is not a law! Using size IV is compatible with all analysis software. Moreover, those patients are well trained in VF testing, and looking at their new test results may improve their depression and encourage their compliance with medications (Figure 12).

**Differential diagnosis of glaucomatous visual field defects**

**Tilted discs.** These usually give superior arcuate defects similar to those produced in glaucoma. However, they are peripheral, and not related to the blind spot. If on the other hand glaucoma developed in an eye with a tilted disc, the resulting defects relate to the blind spot, and are central. Careful inspection of the optic disc will allow for correct interpretation of the field results.

**Central scotoma due to optic neuritis.** This is a papillo-macular bundle defect that crosses the horizontal line. It is associated with reduced visual acuity, and reduced colour vision. It regresses with treatment, but remains central.

**Bitemporal hemianopic defect due to chiasmal lesions.** These can easily be confused with glaucoma, especially if the two pathologies coexist in the same patient (glaucoma plus pituitary tumour) (Figure 13).

**Figure 13a:**
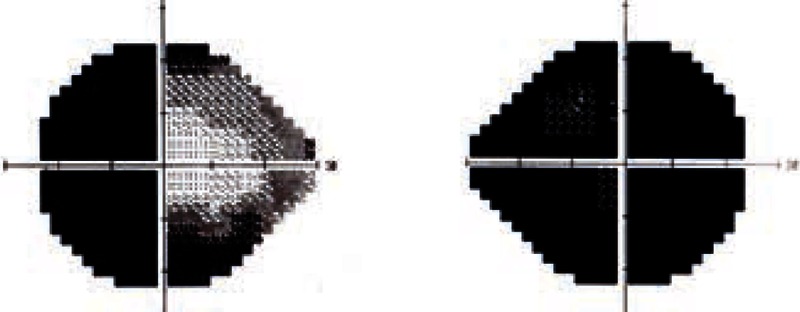
These are the right and left visual fields of a 61 year-old patient, who was diagnosed with POAG 15 years prior to presentation. Both optic discs were terminally cupped. The IOP was poorly controlled in both eyes; however, the patient was aware that his right eye was worse than the left eye.

Looking at the VF of the left eye, there is a clear temporal field defect respecting the vertical midline.

**Figure 13b:**
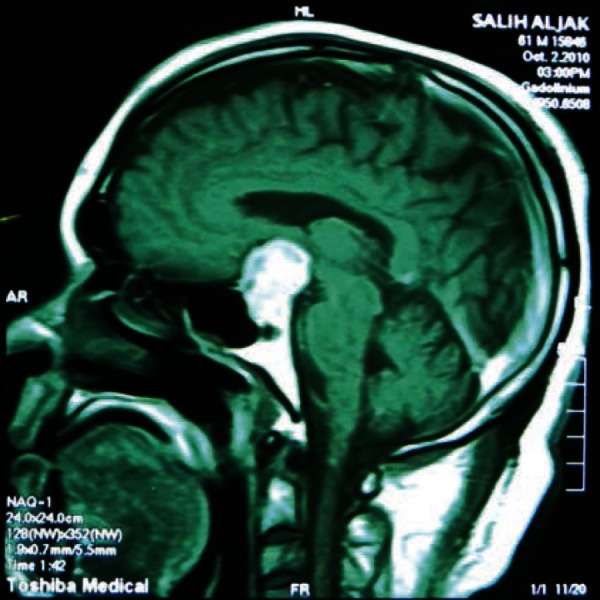
An MRI revealed a large pituitary adenoma responsible for visual loss in both eyes (on top of his POAG).

